# Enhancing pharmacology learning through simulation-based training versus traditional teaching: a quasi-experimental study among undergraduate medical students

**DOI:** 10.3389/fphar.2026.1818874

**Published:** 2026-04-28

**Authors:** Sonali Suryawanshi, Pallawi Khatavkar, Vasundhara Londhe, Gayatri Godbole, Priti Dhande

**Affiliations:** Department of Pharmacology, Bharati Vidyapeeth Deemed to be University Medical College, Pune, India

**Keywords:** clinical reasoning, feedback, knowledge retention, pharmacology, post-test, simulation based learning

## Abstract

**Background:**

Traditional pharmacology tutorials may limit clinical reasoning development in managing life-threatening conditions. Although high-fidelity simulation offers realistic learning, little is known about its comparative effectiveness in teaching pharmacology to undergraduate students.

**Methodology:**

A quasi-experimental study compared traditional tutorials with simulation-based practical among second-year MBBS students. Identical lecture on Organo-Phosphorus Poisoning (OPP) preceded group-specific interventions. Knowledge assessed via MCQs at three time points: pre-test (post-lecture), immediate post-test, delayed post-test (4 weeks). A standardized 5-point Likert-scale questionnaire was used to assess student perceptions. Knowledge scores reported as median (IQR); perceptions (5-point Likert) as n (%). Wilcoxon rank-sum tests compared pre-test, immediate post-test, and 4-week post-test scores between simulation and tutorial groups to assess baseline comparability and intervention effects.

**Result:**

All 126 second-year MBBS students completed baseline assessment post-didactic lecture, with comparable pre-test scores between simulation and tutorial groups [median (IQR): 7 (5–9) vs. 6 (5–7)]. Post-intervention, both groups improved, but simulation group students achieved significantly higher immediate post-test scores and 4-weeks post-test score [r = 0.45]. Three months’ post-intervention, blinded evaluation of a 5-mark exam question on the management of OPP showed higher pass percentage in the simulation group when compared with the traditional teaching group (73.5% vs. 68%; p < 0.05). Student feedback corroborated these results, with majority of simulation students agreeing that their sessions enhanced clinical understanding, engagement, and exam preparedness.

**Conclusion:**

High-fidelity simulation performs noticeably better than traditional tutorials in knowledge acquisition, retention, and student perceptions of clinical relevance for critical cases management like OPP. Simulation fosters improved clinical reasoning, physiological understanding, and examination preparation among medical undergraduates. Pharmacology curricula should try to include simulation-based practicals to bridge theory-practice gaps in clinical pharmacology education.

## Introduction

Simulation-based learning (SBL) has emerged as an important pedagogical strategy in medical education, offering learners an opportunity to engage with realistic clinical scenarios in a controlled, risk-free environment (Arcoraci et al., 2019). Traditional lectures form the foundational cognitive background for medical students, but they often fall short in helping learners translate pharmacological concepts into clinical reasoning and decision-making. In addition to understanding pharmacodynamics, pharmacokinetics, and toxicological principles, students must learn to interpret vital signs, recognize evolving clinical conditions, and link these observations to appropriate therapeutic decisions. This requires not only theoretical knowledge but also the development of applied clinical reasoning skills essential for long-term retention and effective patient care (Gwee, 2009; Corrao et al., 2009).

Unlike traditional lecture-based or tutorial-based formats, SBL integrates experiential, immersive elements that allow students to observe, analyze, and apply pharmacological principles within dynamic clinical contexts (Corrao et al., 2009; Pereira et al., 2019; Issenberg et al., 2005). High- and low-fidelity simulators have been shown to improve knowledge acquisition, enhance clinical reasoning, strengthen communication and teamwork skills, and support the safe practice of critical interventions without jeopardizing patient safety (Pamela et al., 2006).

Evidence from recent studies indicates that SBL not only increases learner engagement but also contributes to deeper understanding and better retention of key pharmacological concepts, including management of emergencies and rare clinical situations (Fodale et al., 2015; McGaghie et al., 2014; McGaghie et al., 2011). Despite its growing acceptance, evidence on integrating simulation techniques into pharmacology education remains limited, with few comparative studies evaluating simulation against traditional teaching approaches (Arcoraci et al., 2019; Hassan et al., 2010; Seropian et al., 2007; Via et al., 2004).

Learners consistently report that simulation offers a richer, more engaging learning experience and facilitates better recall compared to traditional methods (Pereira et al., 2019). In pharmacology, conditions like organophosphorus poisoning and drug antagonism demand quick recognition of clinical signs and prompt administration of antidotal therapy. Lectures alone often fall short in developing these skills.

Simulation enables students to observe evolving clinical signs, monitor physiological changes, and understand the management sequence step-by-step, effectively bridging theoretical knowledge with practical application (Weller, 2004). Against this background, the current study aimed to determine whether simulation-based practical sessions on organophosphorus (OP) poisoning and drug antagonism achieve enhanced learning outcomes and long-term retention compared to traditional tutorial-based teaching.

## Methodology

Ethical approval for the study was obtained from the Institutional Ethics Committee (IEC REF: BVDU/IEC/397/25-26). Participation was voluntary and written informed consent was obtained from all participating students, with participant confidentiality upheld throughout.

This prospective, comparative quasi-experimental study was conducted to evaluate the effectiveness of a simulation-based practical session compared with a traditional tutorial in enhancing immediate learning and long-term retention of pharmacology concepts related to organophosphorus poisoning management and drug antagonism among all second-year MBBS students. This was the first simulation based case scenario activity for the participant students. 126 students attended a 50‐minute standardized lecture from the same faculty member, followed by an identical MCQ-based pre-test to establish baseline knowledge score. Students were then assigned to two parallel groups based on their roll numbers: one engaged in a structured tutorial based on case-discussion, and the other participated in a SimMan-based practical session demonstrating clinical features and stepwise management. Utilizing the high-fidelity simulator in the simulation lab of our institute [Society for Simulation in Healthcare, USA (SSH) accredited], an expert helped set up and manage the organophosphorus poisoning case scenario.

### Teaching intervention

The simulation based intervention involved two SimMan stations conducted sequentially, accommodating approximately 12 students per batch (two subgroups). Each simulation session lasted 25–30 min and included a pre-designed case scenario on organophosphorus poisoning. During the simulated clinical scenario, students watched and examined changing vital indicators, including airway status, breathing pattern, pulse rate, blood pressure, and secretions.

The tutorial-based sessions were conducted in students divided into three groups of approximately 14 students each. These sessions were conducted over 1 h and were case-based and interactive in nature, involving theoretical discussion of the same topic (organophosphorus poisoning), including clinical features and management.

Both interventions were aligned with the same learning objectives and were led by trained pharmacology faculty to ensure uniformity in content delivery, learning objectives, and facilitation approach. The focus of the sessions was on identifying typical signs and symptoms, using clinical reasoning, and managing the condition step-by-step, including airway support and the use of the right medications. A structured debriefing session followed both the interventions, focusing on clarification of clinical reasoning, pharmacological principles, and addressing student queries. Immediate post-test assessment was conducted after completion of the session.

The primary outcome was immediate post-test score. Secondary outcomes included 4-week retention, 3-month examination performance, and student perception. Outcomes were evaluated using the same MCQ instrument immediately post-session (for immediate learning gains) and after 4 weeks (for sustained retention).

Long-term (approximately 3 months’ post-lecture) knowledge retention was evaluated by including a five-mark short-answer question on organophosphorus poisoning management in the first-term end exam. This parameter was calculated for only those students who had attended the intervention, 47 students for simulation and 42 for tutorial session respectively. Faculty graders, unaware of students’ prior group allocation (simulation vs. tutorial), evaluated the responses of students. 50% marks in the five-mark question was considered as pass percentage for that question.

### Learning evaluation

An identical 11-item Multiple-Choice Questionnaire (MCQ) on organophosphorus poisoning and drug antagonism was used to assess immediate learning and long-term retention at three time points: pre-test immediately post-lecture, post-test 1 immediately after the assigned intervention (traditional tutorial or simulation-based practical), and post-test 2 at 4 weeks. Students had 15 min to complete the questionnaire at each assessment.

In order to reduce bias from external learning sources, students received briefing about the test only 10 min prior to each questionnaire administration. The order of the 11 questions was randomized for every test instance. Students remained blinded to their prior test performance. Scores from the three evaluation sessions were recorded, with one point given for each right response and zero for each wrong one.

### Questionnaire content

Content validation of the multiple-choice questionnaire was conducted by a panel comprising of two senior pharmacology faculty, an intensivist experienced in OP poisoning management, and a medical education specialist. Each expert independently rated items for accuracy, relevance, clarity, and alignment with learning objectives; their feedback refined the final instrument. Inter-rater agreement derived from expert ratings showed more than 80% consensus, confirming strong content validity. The questionnaire was pilot-tested on second-year MBBS students from supplementary batch not included in the main study to ensure clarity, comprehensibility, and timing suitability. Moreover, a statistician evaluated its structure and scoring for repeated assessments.

### Statistical analysis

Data were entered and SPSS version 29.0 was used to analyze the data (IBM Corp., Armonk, NY). Test scores were described using descriptive statistics and expressed as median with interquartile range (IQR), while student perception data collected on a 5-point Likert scale were presented as frequencies and percentages [ n (%)]. Repeated measure ANOVA followed by Wilcoxon signed-rank test was used for between‐groups comparison of pre-test, immediate post-test and 4 weeks’ post-test scores.

## Results

Following consent, all second-year MBBS students agreed to participate in the study, and a total of 126 participants were subsequently assigned to either the traditional tutorial group (n = 63) or the simulation-based practical group (n = 63) based on their roll numbers at the day of intervention. The pre-test was given to the entire class immediately after the lecture, baseline scores were comparable between the two groups (Table 1).

**TABLE 1:** Comparison of multiple choice question (MCQ) scores between simulation and tutorial groups at different assessment time points of the study.

Study groups	Pre-test score	Immediate post-test score	4 weeks follow-up score	p value	Effect size (r)
Simulation-based practical group	7 (5–9)	9 (9–10)	10 (8–10)	0.001	0.45
Traditional tutorial group	6 (5–7)	7 (5–10)	7 (5–10)	0.53	0.2

Repeated measure ANOVA followed by Wilcoxon signed-rank test for between‐groups comparison.

Attendance varied across the study phases. During the intervention sessions, 47 students were present in the simulation-based practical group and 42 students in the traditional tutorial group. At the 4-week follow-up assessment, 47 students from the simulation group and 39 students from the tutorial group were present and completed the assessment. Statistical analyses were performed based on the students who were present at each respective assessment point.

Table 1 depicts that the median pretest score in simulation method was increased from 7 to 9 at immediate posttest and further increased to 10 after 4 weeks (overall effect size r = 0.45), while in tutorial method it was increased from 6 to 7 at immediate posttest and remained unchanged after 4 weeks (overall effect size r = 0.2).

**FIGURE 1:**
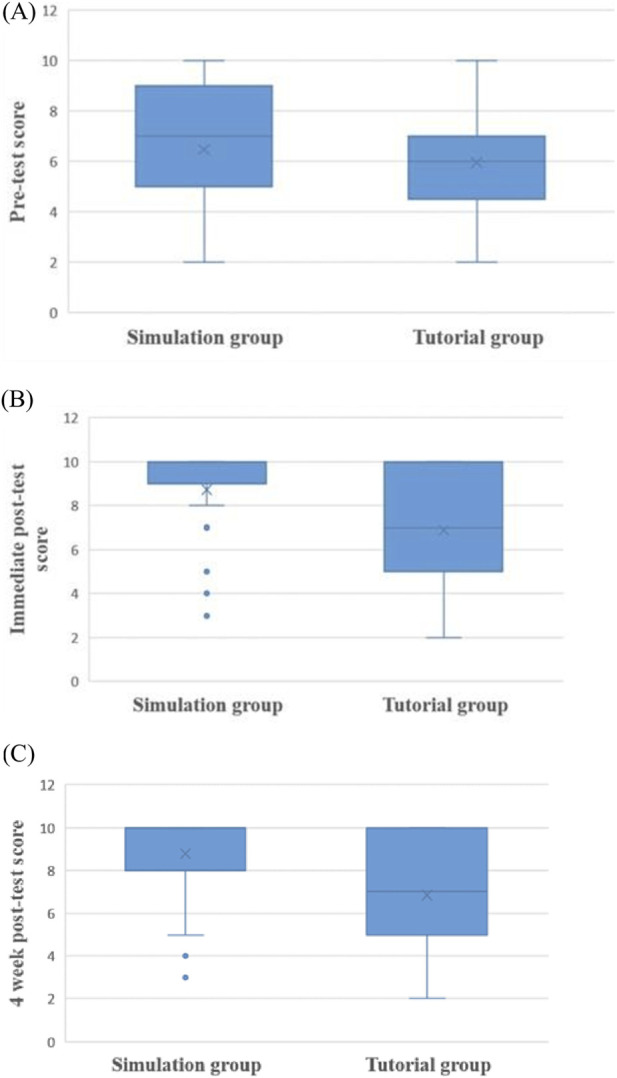
Distribution of knowledge scores across assessment time points [pre-test **(A)**, immediate post-test **(B)**, 4 weeks’ post-test **(C)**] in simulation-based practical and traditional tutorial groups.

Pre-test baseline scores were comparable between the simulation and tutorial groups, confirming initial equivalent knowledge levels post-lecture (Figure 1A). Both groups showed score improvements post-intervention; however, the simulation group achieved a significantly greater gain immediately following the post intervention (P < 0.001) (Figure 1B). This improvement was maintained at delayed 4 weeks post-testing, with simulation students scoring significantly higher (P < 0.001) (Figure 1C), suggesting better retention of pharmacological concepts. Boxplots illustrate these patterns, revealing elevated median scores and greater consistent performance in the simulation group across all assessment points.

Student feedback clarified that around 88% students in both the intervention groups found the sessions extremely beneficial for clinical understanding of the symptoms of Organophosphorus poisoning as well as its monitoring and treatment. Comparing the feedback it was evident that students from simulation-based practical group felt more encouraged to ask questions and clarify their doubts (95.24% vs. 78.57%) as well as found the session beneficial in examination preparation (92.9% vs. 76.18%) (Tables 2, 3).

**TABLE 2:** Student feedback on simulation-based practical conducted on organophosphorus poisoning.

Questions	Response n = 42				
	Strongly disagree n (%)	Disagree n (%)	Neutral n (%)	Agree n (%)	Strongly agree n (%)
The Sim-Man scenario felt realistic and improved my clinical reasoning skills	0	0	5 (11.90)	17 (40.48)	20 (47.62)
Observing the vital signs and management steps (airway, breathing, pulse, BP, secretions) during the simulation helped me to understand the signs, symptoms and management of OP poisoning	0	1 (2.38)	4 (9.52)	15 (35.71)	22 (52.38)
I felt encouraged to ask questions and clarify my doubts during simulation based practical	0	0	2 (4.76)	17 (40.48)	23 (54.76)
The debriefing session after simulation helped consolidate my learning	0	2 (4.76)	5 (11.90)	18 (42.86)	17 (40.48)
Sim-Man session will help me write theory answers and perform better in viva	0	0	3 (7.14)	18 (42.86)	21 (50)

**TABLE 3:** Student feedback on traditional tutorial session conducted on organophosphorus poisoning.

Questions	Response n = 42				
	Strongly disagree n (%)	Disagree n (%)	Neutral n (%)	Agree n (%)	Strongly agree n (%)
The tutorial session improved my understanding of the signs, symptoms, and immediate management of OPP	0 (0)	0 (0)	5 (11.90)	15 (35.71)	22 (52.38)
Tutorial session improved my understanding and retention of pharmacological concepts related to organophosphorus poisoning	2 (4.76)	2 (4.76)	6 (14.28)	22 (52.38)	10 (23.80)
I felt encouraged to ask questions and clarify my doubts	2 (4.76)	2 (4.76)	5 (11.90)	22 (52.38)	11 (26.19)
Tutorial session will help me write theory answers and perform better in viva	2 (4.76)	2 (4.76)	6 (14.28)	20 (47.61)	12 (28.57)

Three months post-intervention when the performance on the term-end exam question evaluating organophosphorus poisoning management was assessed, it was found that more tutorial-group students left this optional question unanswered (7 in the tutorial group vs. 3 in the simulation-based group) and the pass percentage for this question was higher in the simulation-based group (73.47%) than in the tutorial group (68%) (p > 0.05).

## Discussion

Simulation-based learning (SBL) is increasingly applied across various healthcare domains to ensure patient safety and strengthen learners’ technical competencies. In pharmacology education, its use is also becoming popular, as it is believed to enhance learning, retention, and conceptual breadth beyond traditional lectures (Arcoraci et al., 2019; Cooke et al., 2017).

The present study investigated whether incorporation of simulation-based practical session, following a didactic lecture, offers a greater improvement in learning and long-term retention of pharmacology concepts compared to a traditional tutorial among second-year MBBS students. In this study, the pre-test conducted immediately after the didactic lecture revealed comparable baseline scores between groups, confirming that the lecture successfully imparted uniform foundational knowledge in pharmacology across all the class students. These results underscore the vital role of didactic lectures in conveying core theoretical concepts in pharmacology and equalizing participants’ initial knowledge for subsequent instructional interventions (Arcoraci et al., 2019). Although 126 students were enrolled, attendance dropped substantially at the intervention and follow-up phases. Attrition occurred across different phases of the study, which is not uncommon in educational interventions conducted within routine academic schedules. The analysis was therefore performed on students who were present at each respective assessment (per-protocol analysis), as meaningful evaluation of the intervention required exposure to the respective intervention session.

High-fidelity simulation has been shown to benefit medical, nursing, postgraduate students, and residents in earlier research studies (Aqel and Ahmad, 2014; Varley et al., 2015). In the Indian setting, organophosphorus poisoning is a clinically significant and frequently encountered emergency. Its management necessitates the integration of pharmacological knowledge with clinical decision-making, which makes it well suited for simulation-based learning. The choice was also influenced by previous teaching experience, which showed that students frequently struggle to apply this subject in clinical settings using conventional techniques. In line with this, our research revealed that students who participated in high-fidelity simulation provided noticeably more accurate answers to the Multiple Choice Question (MCQ) test than the traditional tutorial group in both immediate post-test and post 4-week assessment (Table 1) which we conducted on the topic Organophosphorus poisoning used for this study. The scores of students from the simulation group were significantly better than that of the traditional tutorial group (r = 0.45).

While gaining knowledge is crucial, retaining it over time is equally vital. Multiple studies have demonstrated significant declines in knowledge and skills within 3 months absent reinforcement. (Aqel and Ahmad, 2014; Nimbalkar et al., 2015). Atrey M et al. reported strong outcomes from SBL, including improved retention (96.8%), higher scores, and better application of theoretical knowledge to practical scenarios; it proved particularly effective in experimental pharmacology without requiring animal use (Atray et al., 2017) whereas the study by Kasturi R et al. does not support enhanced long-term retention with simulation versus traditional facilitator guided paper based problem solving sessions. (Kasturi et al., 2009). Findings from the present study align with Pereira et al. (2019), and from Ennen and Satin (2010), where simulation-based learning (SBL) aided retention of pharmacological correlation with clinical case scenario (Organophosphorus poisoning) which has been very well depicted in the Figures 1B,C.

Although the 3-month post-intervention assessment did not demonstrate a statistically significant difference between the groups (p > 0.05), the observed pattern of scores may suggest a possible trend toward improved application of knowledge in the simulation group. Overall, while simulation-based teaching showed clear benefits in immediate learning and short-term retention, its impact on long-term summative performance could not be conclusively established in the present study.

Our findings align with developing international evidence supporting role of simulation in enhancing pharmacology education among undergraduate medical students (Robinson et al., 2011; Mieure et al., 2010; Seybert et al., 2006). The positive observations obtained in this study reflect the realism of high-fidelity mannequins which “talk, breathe, and blink” bringing students closer to authentic clinical scenarios, as noted in prior studies (Founds et al., 2011; Baptista et al., 2014). It is not necessary to use costly and sophisticated simulators for such training but studies have shown that low cost, accessible simulation methods like patient simulator or PC simulator, have also produced enhanced learning and performance (Pereira et al., 2019; Hassan et al., 2010; Agostino et al., 2025).

Where Chakravarthy et al. (2011), reported gains in medical knowledge, confidence, and subject understanding during emergency rotations with simulation based education, present study found that students from both simulation-based practical sessions as well as tutorial session reported enhanced clinical comprehension. At the same time encouragement to ask questions, clarifying doubts and exam preparedness was comparatively better in the simulation group (Tables 2, 3).

These findings confirm that SBL in pharmacology practicals is well accepted by medical undergraduates, markedly improving their clinical reasoning, grasp of physiological concepts, and self confidence in handling critical scenarios such as organophosphorus poisoning. However, larger, multicentric studies are needed to assess the long-term impact of simulation interventions on knowledge retention and skill acquisition.

## Strengths and limitations

This study features key strengths, including a clinically relevant simulation-based practical in undergraduate pharmacology and multiple assessments evaluating immediate gains and long-term retention. Baseline pre-test scores were comparable between both the groups and performance in this study related tests did not affect their academic progress.

Limitations include the quasi-experimental design and single-center setting, which may limit generalizability; unassessed prior academic performance poses a potential confound (mitigated by baseline equivalence); and possible informal information sharing among same-batch students. Nonetheless, the findings provide preliminary evidence for simulation-based learning benefits in pharmacology education.

Formal reliability testing (e.g., Cronbach’s alpha) was not performed for the Multiple-Choice Questionnaire used in the study. However, the questionnaire was reviewed by a statistician for structural consistency and suitability for repeated assessments.

Implementation faces hurdles too: The transition to Competency-Based Medical Education (CBME) in India has reduced second-year MBBS duration from 1.5 years to 10 months, requiring educators to cover a wide curriculum in less time. Simulations require extensive preparation and expert facilitation, making time the biggest obstacle, along with some pharmacology topics that do not fit this format well.

## Conclusion

In this study, a simulation-based practical enabled undergraduates to link organophosphorus poisoning to clinical practice via real-time physiological observations and interactive management discussions, fostering deeper engagement than traditional tutorials. While both approaches boosted immediate performance, the simulation group demonstrated superior long-term retention of complex principles. Simulations allow safe observation of multisystem effects and drug outcomes challenging to replicate in lectures despite requiring greater faculty and institutional resources. These results support integrating structured simulations to bridge the theory-practice gap in undergraduate pharmacology teaching.

## Data Availability

The raw data supporting the conclusions of this article will be made available by the authors, without undue reservation.
